# The Loud Bill

**Published:** 1898-03

**Authors:** 


THE LOUD BILL.—The JOURNAL notes with great satisfaction the defeat in Congress of the infamous Loud Bill. It will be remembered that this bill aimed at the extinction of all the weaker publications in as much as, had it become a law, the postage on second-class matter would have been increased to the extent of prohibition. It would also have cut off all "sample copies" of publications of the character of the JOURNAL,—the principal means relied upon by publishers for securing new subscribers, and it would have converted every office of publication into a postoffice. It would have required the publisher of every newspaper and journal and magazine to sort out its mail in separate packages, and thus relieved the postmaster of half his work.

It is believed that there was a "bug under that chip;" that it was attempted in the interests of some big publishing trust, and designed to drive out competition.

